# Preparation and Application of Nanostructured ZnO in Radiation Detection

**DOI:** 10.3390/ma17143549

**Published:** 2024-07-18

**Authors:** Jingkun Chen, Xuechun Yang, Yuandong Ning, Xue Yang, Yifei Huang, Zeqing Zhang, Jian Tang, Pu Zheng, Jie Yan, Jingtai Zhao, Qianli Li

**Affiliations:** 1 School of Materials Science and Engineering, Shanghai University, Shanghai 200444, China; 2 School of Environmental and Chemical Engineering, Shanghai University, Shanghai 200444, China; 3 School of Materials Science and Engineering, Guilin University of Electronic Technology, Guilin 541004, China; 4 Institute of Nuclear Physics and Chemistry, Chinese Academy of Engineering Physics, Mianyang 621022, China

**Keywords:** radiation detection, scintillator, nanostructured ZnO, ultrafast, X-ray imaging

## Abstract

In order to adapt to the rapid development of high-speed imaging technology in recent years, it is very important to develop scintillators with an ultrafast time response. Because of its radiation-induced ultrafast decay time, ZnO has become an important material for radiation detection and dosimetry. According to different detection sources and application scenarios, ZnO is used in various radiation detectors in different structures, including nanoarrays and nanocomposites. In this paper, the synthesis methods and research status of various nanostructured ZnO-based materials and their applications in the detection of high-energy rays (X-rays, γ-rays) and high-energy particles (α, β and neutron) are reviewed. The performance discussion mainly includes spatial resolution, decay time and detection efficiency.

## 1. Introduction

When passing through the medium, high-energy rays and particles will interact with the electrons/nuclei of the medium atoms, resulting in secondary particles (visible light and electrons, etc.) that can be easily recorded, or voltage pulse signals are generated at the probe of the detector. A radiation detector is used to analyze the energy and intensity of the radiation source by collecting secondary particles or voltage pulse signals. According to the material type, the radiation detector can be categorized as a gas detector, semiconductor detector or scintillator detector. Gas detectors such as ionization chambers, proportional counters and Geiger counters are widely used in measuring instruments, pocket dosimeters and area monitors. Semiconductor detectors such as silicon surface barrier detectors, HPGe and cadmium zinc telluride are used for α particles and γ ray radiation spectra. A scintillator is a kind of material that converts radiation energy into visible or ultraviolet light. ZnS and CdWO_4_ are among the first generation of scintillators. With the advent of photomultiplier tubes in 1940, several scintillators have been developed successively, among which NaI:Tl is the most commonly used scintillator so far. Its production often requires specific and expensive equipment, as well as long and complex procedures associated with producing crystal growth.

Since the 21st century, there has been rapid development of radiation detectors in the fields of medical imaging, high-energy and nuclear physics, oil exploration and other applications. People’s demands for the performance of detectors, such as time and spatial resolution, are increasing. For instance, the requirements for spatial resolution in nanofocused beams used in synchrotron radiation are particularly stringent. PET-CT requires temporal resolution [1,2,3]. XFEL [4] uses ultra-short pulse durations to measure the dynamics of matter, thus X-ray detectors with ultra-fast time-resolution are needed. Hu et al. [5,6,7] suggest that ZnO:Ga and BaF_2_:Y are both considered for hard X-ray imaging applications at kilohertz. For BaF_2_:Y, its advantages lie in the fast decay time of luminescence and the maturity of large-size crystal preparation technology. However, BaF_2_:Y crystal has two scintillation luminous components, and the ultrafast luminous components of BaF_2_: Y account for a relatively small proportion. Moreover, the emission peak in the deep ultraviolet region (220 nm) is not conducive to matching with existing photodetectors [8]. The radiation exciton decay of ZnO has a fast scintillation response (sub-nanosecond emission lifetime), and ZnO also has good scintillation performance. ZnO is a direct band gap (3.37 eV) semiconductor with a large exciton binding energy (60 meV). Its high density (5.7 g/cm^3^) and effective atomic number (28) ensure sufficient radiation cutoff ability. In addition, ZnO-based materials also have a high melting point (1975 °C), non-deliquescence, chemical stability and strong radiation hardness [9]. Based on these advantages, ZnO with various structures has been used in various radiation detectors.

According to the application of different detectors and scenarios, ZnO morphology and preparation methods are also different. Currently, ZnO materials used for radiation detection include single-crystal, thin-film, transparent ceramics and nanostructures [10,11,12,13], and the preparation methods include electrodeposition, hydrothermal, sol-gel and chemical methods [14,15,16,17]. Every preparation method has its own advantages; for example, the coprecipitation method has the advantages of large synthesis scale at room temperature, simple experimental equipment, easy doping impurities and low cost [18,19]. The magnetron sputtering method has the advantages of high deposition rate, high power efficiency, low substrate temperature and small substrate damage [20]. Compared with the morphology of bulk crystals and transparent ceramics, nanostructured ZnO has better optical properties [11,21,22]. Nano-scale ZnO materials can reduce the self-absorption of ZnO materials. Venevtsev et al. [12] studied and compared scintillation properties of ZnO samples with different morphology, including whiskers, nanowalls and ceramics. The results show that the total light transmittance, photoluminescence and radioluminescence spectra and radioluminescence kinetics vary significantly with the sample structure and preparation conditions. Doping is an effective method to improve the scintillation performance of ZnO-based materials [10,23,24,25,26]. By optimizing different parameters such as dopant type, concentration and doping mode to reduce the trapping center, better optical performance is obtained. Elements such as Al, Ga and In are commonly used as N-type dopants for ZnO growth, because they directly replace Zn ions and occupy Zn vacancy. Among them, Ga has been widely studied because of its low reactivity and nearly perfect match with Zn. In addition, Ga, as a shallow donor level, can provide enough electrons for photoelectric properties [27].

**Figure 1:**
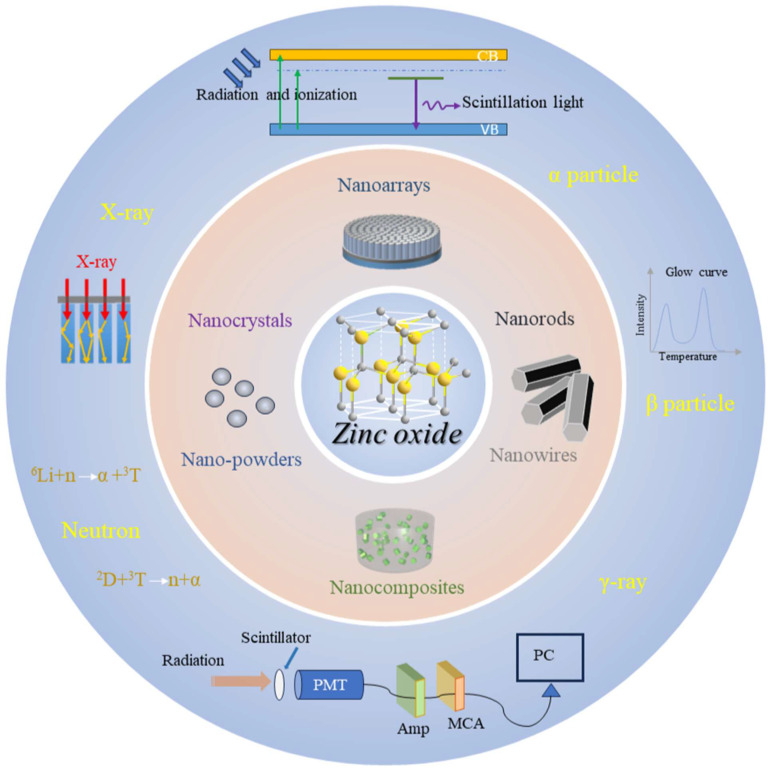
ZnO nanostructures and radiation detection applications.

In this paper, the preparation methods and research status of ZnO-based materials with different nanostructures in the field of radiation detection in recent years are introduced, and the detection performance and device application of ZnO are summarized, which provides a reference for further research and application of ZnO in the field of radiation detection. As shown in Figure 1, this paper includes the morphology of nanostructured ZnO-based materials such as nanorods/wires, nanoarrays, nanopowders/crystals and nano-composites and their detection of different radiation sources.

## 2. Nanostructured ZnO

ZnO is generally wurtzite in structure, and can be transformed into sphalerite under high pressure. In the wurtzite structure, each Zn atom is surrounded by four O atoms, forming a tetrahedral structure, and vice versa. At the nanoscale, the large surface area to volume ratio and size reduction of the particles lead to atom-like behavior of nanostructured ZnO, and affect the electronic states between conduction and valence bands due to the high quantum confinement effect. In addition, because the (002) crystal face has a relatively high surface energy, in order to minimize the free surface energy, ZnO has a *c*-axis-preferred growth characteristic; that is, ZnO crystals usually grow along the (002) crystal face [28]. Of course, the surface energy of the (002) crystal surface can be reduced by changing the growth method and conditions and doping, and the crystal growth along the (100) plane can be promoted, and vice versa [29]. Using this property, vertically aligned nanorods/wires can be grown on the substrate. Such nanoarrays are a common form of ZnO used in radiation detection, and ZnO is often combined with polymers to form composite films to expand its application scenarios.

### 2.1. Nanorods/Wires

**Figure 2:**
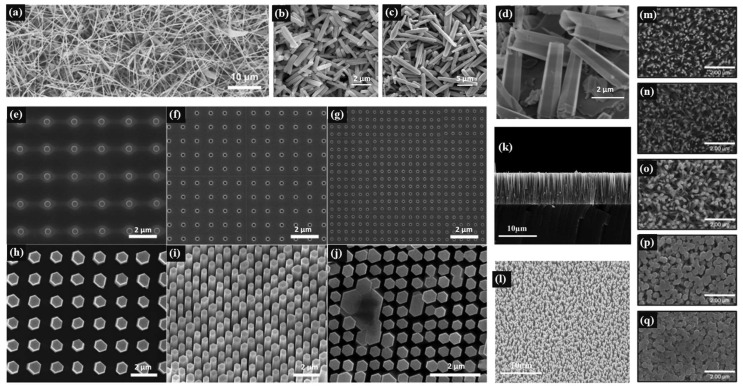
(**a**) SEM images of ZnO nanowires [30]. FE-SEM images of Ga (**b**) and In (**c**)-doped ZnO nanorods [34]. (**d**) SEM images of ZnO:Mo (10%) [36]. SEM images of two-dimensional periodic growth hole patterns with different sizes and periods: (**e**) ~410 nm, 2 μm, (**f**) ~200 nm, 1 μm, (**g**) ~140 nm, 0.5 μm, and ZnO nanowire arrays: (**h**–**j**) corresponding to (**e**–**g**), respectively [31]. FE-SEM images of (**k**) cross-sectional and (**l**) surface views for the ZnO:Ga nanorod arrays [40]. Plan-view SEM images of ZnO nanorods fabricated using (**m**) 3:1, (**n**) 2:1, (**o**) 1:1, (**p**) 1:2, and (**q**) 1:3 HMTA and ZnAc concentration ratios [43].

The main preparation methods of ZnO nanorods/wires are hydrothermal, chemical vapor transport and vacuum thermal evaporation. Among them, the vacuum thermal evaporation method is a very simple method to prepare nanowires. Table 1 shows the preparation methods and peculiarities of ZnO-based materials with nanowires/rods. Butanovs et al. [30] loaded the mixture of ZnO and carbon powder 1:1 into a quartz tube and stored it at 950 °C for 90 min. The smooth nanowires with a diameter of about 100 nm were then naturally cooled to room temperature with a length of 10–100 μm (Figure 2a). The main advantage of the hydrothermal method is that high-quality nanostructured ZnO can be synthesized at a relatively low temperature [15]. In order to facilitate large-scale and low-cost manufacturing, the morphology parameters of nanorods/wires can be controlled by adjusting the concentration of precursor, reaction temperature and PH, and the type and concentration of doped ions (Ga, In and Mo, etc.) can be conveniently adjusted to prepare more uniformly doped ZnO-based nanorods [31,32]. However, because the nanorods/wires prepared by the hydrothermal method will be accompanied by the formation of oxygen vacancy and other defects, improving the synthesis quality must be considered. Hu et al. [29] found that by adjusting the concentration of In doping, different concentrations of In doping can promote or inhibit the (002) surface of ZnO bars, respectively. Buryi et al. [26] also found that Er acts as an inhibitor for rod growth when the concentration of doping element Er in the hydrothermal ZnO:Er is low, and acts as a catalyst when the doping concentration is increased.

**Table 1:** Preparation methods and peculiarities of ZnO-based materials with nanowires/rods.

Morphology	Material Types	Preparation Methods	Peculiarities	Reference
Nanowire	ZnO	Atmospheric pressure chemical vapor transport method	The length is 10–100 μm and the diameter is 100 nm	[30]
Nanowire	ZnO	Vacuum thermal evaporation; Thermal oxidation process	The average diameter of ZnO nanowires formed in zinc films with thicknesses of 125, 250 and 500 nm is 50, 75 and 83 nm, respectively	[33]
Nanorod	ZnO:Ga/ZnO:In	Hydrothermal	Ga and In-doped nanorods annealed at 750 °C and 500 °C respectively have better crystal quality (Figure 2b,c)	[34]
Nanorod	ZnO:Mo	Hydrothermal	/	[25,35,36,37]
Nanorod	ZnO:Er	Hydrothermal	The morphology of ZnO:Er changes from undoped nanorods to microrods	[26]

### 2.2. Nanoarrays

ZnO nanoarrays generally refer to vertically arranged nanorods/wires. Due to the optimal growth characteristics of ZnO on the *c*-axis, vertically arranged nanorods/wires can be prepared on the substrate by electrodeposition, or a seed layer of a certain thickness can be sputtered on the substrate by magnetron sputtering or spray pyrolysis. Then the seed layer is placed in the precursor solution to grow vertically aligned nanoarrays. The advantage of such a morphology is that it can effectively prevent the transverse diffusion and propagation of light, and the ZnO-based nanorods have a good optical waveguide effect on the scintillation light, so nanoarray ZnO devices have very high spatial resolution. Table 2 shows the preparation methods and peculiarities of ZnO-based materials with nanoarrays. For nanoarrays prepared by electrodeposition, the choice of substrate will directly affect the lattice mismatch of the nanoarray. Masakazu et al. [38] chose ZnO:Ga/sodium-calcium glass as the substrate, and the lattice mismatch rate of the prepared ZnO-VNWS was almost 0. Izaki et al. [14] prepared (002) oriented ZnO-VNWS on <111>-Au/Si wafers and <0001>-ZnO:Ga substrates, with estimated lattice mismatches of 12.9–14.8% and 0.4–1.1%. As it has a low mismatch of wurtzite crystal symmetry and lattice constant like ZnO, GaN can also be used as a substrate [31].

For the method of hydrothermal growth of sputtered seed layer in precursor solution, a seed layer with different densities can be prepared by magnetron sputtering or spray pyrolysis, and nanorods/wire arrays with different lengths and widths can be prepared by adjusting different growth conditions. Li et al. [39,40] first grew a 100 nm ZnO seed layer on a quartz substrate by magnetron sputtering, and then added it to a certain proportion of HMTA, zinc nitrate hexahydrate [Zn(NO_3_)_2_·6H_2_O] and gallium nitrate hydrate [Ga(NO_3_)_3_·*x*H_2_O]. The vertically grown ZnO:Ga nanorods array was obtained by hydrothermal heating at 95 °C for 3 h in solution. FE-SEM images of a ZnO:Ga nanorod array are shown in Figure 2k,l. Sinem et al. [41,42] added sodium citrate and ammonium hydroxide (NH_4_OH) to the growth solution as the precursor solution when preparing the nanorod arrays by the hydrothermal method, and controlled the reaction rate by buffer. Angub et al. [43] adjusted the concentration ratio of HMTA to ZnAc to obtain nanorod arrays of different sizes (Figure 2m–q). Muslimov et al. [44] prepared wurtzite ZnO whisker arrays by chemical vapor deposition, and the heights reached to 60 μm. The whiskers were approximately cylindrical and evenly distributed on the substrate surface, but not uniformly arranged. In conclusion, for the preparation of nanoarrays, the advantages of the one-step method (electrodeposition) are simplicity and efficiency, and the advantages of the two-step method (seed layer and hydrothermal method) are controlled preparation and high synthesis quality.

**Table 2:** Preparation methods and peculiarities of ZnO-based materials with nanoarrays.

Morphology	Material Types	Preparation Methods	Peculiarities	Reference
Nanoarray	ZnO	Focused ion beam etching; Hydrothermal	Focused ion beam etching has advantages in making periodic patterns	[31]
Nanoarray	ZnO	Electrodeposition	The length and width of VNWS are estimated to be about 200 and 40 nm, respectively	[38]
Nanoarray	ZnO	Electrodeposition	For Au/Si wafers and ZnO:Ga substrates, the lattice mismatches are 12.9–14.8% and 0.4–1.1%, respectively	[14]
Nanoarray	ZnO	Electrodeposition	The width of ZnO-VNWS is 0.16~0.2 μm and the length is 5.4 μm	[45]
Nanoarray	ZnO	Atomic layer deposition; Hydrothermal	The substrate is BGO	[46]
Nanoarray	ZnO:Ga	Magnetron sputtering method; Hydrothermal	The growth density of ZnO:Ga nanorods is higher than that of ZnO	[39]
Nanoarray	ZnO:Ga	Magnetron sputtering method; Hydrothermal	The average diameter of ZnO:Ga nanorods is about 0.5 μm, and the average length is about 15 μm	[40]
Nanoarray	ZnO/ZnO:Ga	Magnetron sputtering method; Hydrothermal	The lengths (thicknesses) of the vertically arranged nanorods were 15 μm and 17 μm, respectively	[41,42]
Nanoarray	ZnO	Spray pyrolysis method; Hydrothermal	When the concentration of HMTA is low, the length, width and density of hydrothermal ZnO nanorods are shorter	[43]
Nanoarray	ZnO	Chemical vapor deposition	The whiskers are approximately cylindrical, uniformly distributed on the substrate surface, but not uniformly arranged, with a length of 20~60 μm and a diameter of 1~4 μm	[44,47,48]

### 2.3. Nanopowders/Crystals

Nanopowder/crystal ZnO is generally composited with polymers to form thin films rather than directly applied to the device. The sol-gel method, coprecipitation method, hydrothermal method and wet chemical method require simple equipment and a short period of preparation. Table 3 shows the preparation methods and peculiarities of ZnO-based materials with nanopowder/crystal.

**Figure 3:**
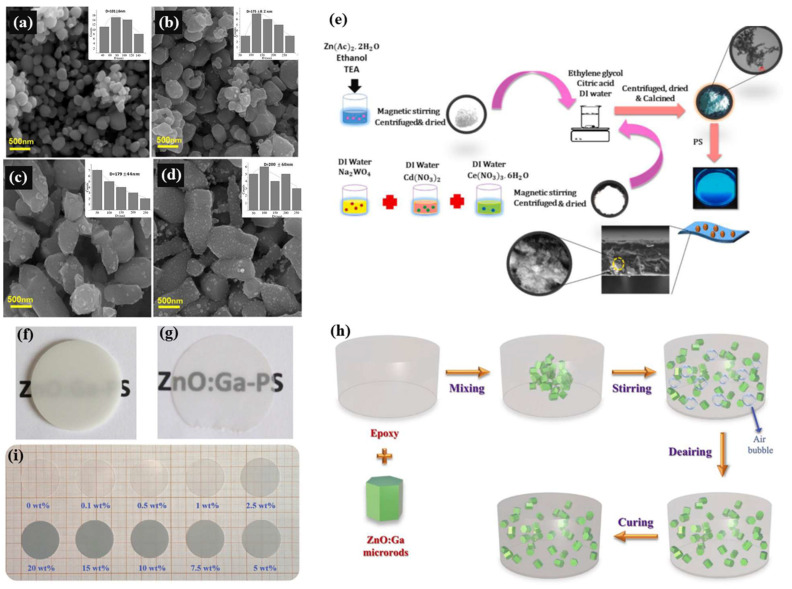
FE-SEM images of (**a**) pure ZnO, (**b**) GZO-0.5 at.%, (**c**) GZO-1 at.%, (**d**) GZO-2 at.% [27]. (**e**) Brief fabrication schematic of the ZnO/CdWO_4_:Ce nanocomposite [17]. (**f**) 1 mm and (**g**) 0.17 mm thick images of ZnO:Ga-PS composites, where the proportion of ZnO:Ga is 10 wt.% [23]. (**h**) Synthesis technology of ZnO:Ga-EP composites [53]. (**i**) Photos of ZnO:Ga-EP composites (0.5 mm thick) with different ZnO:Ga mass percentages [53].

Alamdari et al. [27] obtained ZnO:Ga with different doping concentrations by the sol-gel method. The preparation process is shown in Figure 3a. With the increase of Ga-doping concentration, the particle size of the prepared nanoparticle increases from 101 to 200 nm, and the angle of (101) diffraction peak slightly increases. This is due to the fact that the ionic radius of Ga^3+^ (0.062 nm) is smaller than that of Zn^2+^ (0.074 nm). Mazhdi et al. [49,50] prepared pure ZnO nanoparticles doped with Gd by the coprecipitation method. Gd-doping can effectively affect the size of the nanoparticles. Under low Gd doping concentration, the size of the nanoparticles increases due to nucleation, while under high Gd doping concentration, the size of the nanoparticles decreases due to cation vacancy. Nanopowder ZnO is usually applied by composite polymers.

**Table 3:** Preparation methods and peculiarities of ZnO-based materials with nanopowder/crystal.

Morphology	Material Types	Preparation Methods	Peculiarities	Reference
Nanopowder	ZnO:Ga	Sol-gel method	With TEA as the stabilizer, 1% Ga-doped samples showed better crystallinity	[27]
Nanocrystal	ZnO/ZnO:Gd	Coprecipitation	At low Gd concentration, the size of nanocrystals increases due to nucleation process, while at high Gd concentration, the size of nanocrystals decreases due to cation vacancy	[49,50]
Nanocrystal	ZnO:Ni/Cu	Wet chemical method	/	[51]

### 2.4. Nanocomposites

In order to expand the application field of X-ray detection system, the preparation of ZnO nanopowder by hydrothermal, sol-gel and other methods, and then the compositing of polymer into flexible films is a relatively common research direction. Another reason why the preparation of polymer-based scintillators is of great interest is that they are easier to prepare and have a shorter preparation period than single crystal scintillators [52]. PVA, EP, PS and PDMS are commonly used substrates for preparing such composites. The EP composite film has good optical transmissibility, and PVA and PDMS can be used as the matrix of flexible composite films to develop curved surface imaging and other applications. Table 4 shows the preparation methods and peculiarities of ZnO-based materials with nanocomposite.

Buresova et al. [23] first prepared ZnO:Ga powder by photo-induced precipitation, then calcined it in air and Ar/H_2_ atmosphere and mixed the powder with PS (ZnO:Ga 10 wt.%) into a 1 mm thick film. Samples reduced to 0.17 mm show good transparency (Figure 3f). Li et al. [53] synthesized ZnO:Ga-EP composites by hydrothermal polymerization (Figure 3h,i). Ahmad et al. [54] synthesized a flexible composite film with nanoparticles (CuO, ZnO or both), PVA and glycerol plasticizers. ZnO nanoparticles were synthesized by the solvothermal method with ethanol as the solvent.

**Table 4:** Preparation methods and peculiarities of ZnO-based materials with nanocomposite.

Morphology	Material Types	Preparation Methods	Peculiarities	Reference
Nanocomposite	ZnO:Ga, PS	Photochemical method	Good transparency	[23]
Nanocomposite	ZnO/ZnO:Ga, PS	Sol-gel method; Polymerization method	Flexible ZnO and ZnO:Ga scintillation films were simply and economically prepared	[55,56]
Nanocomposite	ZnO:Ga, EP	Sol-gel method; Polymerization method	The thickness is about 2 mm	[57]
Nanocomposite	ZnO/CdWO_4_:Ce	Sol-gel method; Chemical method	Doped Ce changes the morphology of ZnO/CdWO_4_ from spherical particle structure to nanorods	[17]
Nanocomposite	ZnO:Ga, EP	Hydrothermal; Polymerization method	The preparation is simple and ZnO:Ga is a hexagonal wurtzite structure	[53]
Nanocomposite	CuO/ZnO; PVA; Glycerin plasticizer	Solvothermal method; Solution casting process	The average particle size of ZnO is 8 ± 3 nm; flexibility and feasibility of large-scale preparation	[54]
Nanocomposite	ZnO; poly (styrene-co-acrylic acid)	Solvent casting process	Can be customized into any shape, environmental protection, easy to recycle	[58]

## 3. Application of ZnO in Radiation Detection

**Figure 4:**
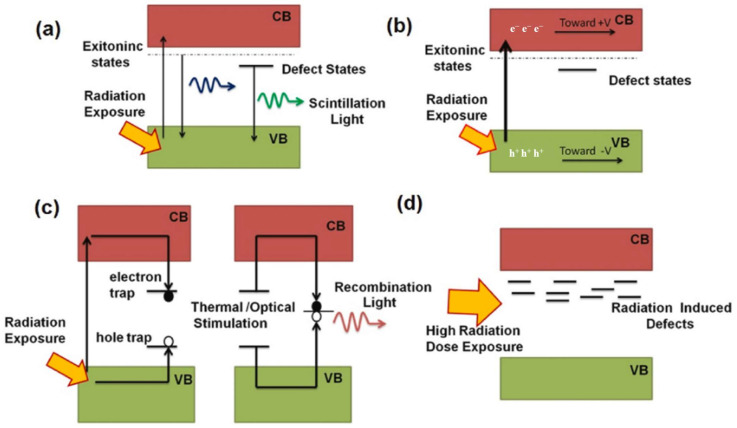
(**a**) Scintillation process, (**b**) ionization process, (**c**) stimulated light dosimetry process and (**d**) radiation-induced defects [59].

According to the different principles used, radiation detectors can be roughly divided into four categories: scintillator detectors, ionization detectors, TL/OSL detectors and radiation damage detectors [59]. As shown in Figure 4a–d, the detection principle of a scintillator is that valence band electrons are excited to the conduction band to form electron hole pairs caused by radiation, and free electrons in the conduction band move to the exciter band and valence band hole recombination by coulomb action to form excitons. Part of the energy of exciton instability deactivation is released by emitting ultraviolet/visible light, which can be measured by a photon detector. Current photon detectors, including PMT, photodiodes and CCD, can be selected according to their different quantum efficiency for different emitted wavelengths of light. The ionization detector is a device for radiation detection by detecting the current generated by the directional movement of the electron hole pair generated by radiation under the action of an applied electric field. Such a medium can be solid, gas, etc. The TL/OSL detector uses heat/light as an excitation source to excite the radiation-generated electron hole pairs trapped by natural/man-made defects, which produce ultraviolet/visible light that can be detected by the photodetector when recombined. The principle of radiation damage detection is to use the effect of radiation to change the structure, morphology, optical, electrical and thermal properties of materials to detect the type and dose of radiation sources.

ZnO has been successfully applied in four types of radiation sensors. Edgars et al. [30] used a ZnO nanowire ionization detector synthesized by atmospheric pressure chemical vapor transport method to achieve linear X-ray measurement. Li et al. [39] prepared hydrothermal ZnO:Ga nanoarrays to achieve ultra-high spatial resolution (1 μm) and ultra-fast time resolution (subnanosecond) for X-rays. Chen et al. [40] prepared a ZnO array scintillation screen to achieve high signal-to-noise ratio detection of α particles. Asemi et al. [60] prepared a PS/BECV-DHF/ZnO sample, which showed high counts up to 3300 CPM/μC of ^60^Co (γ-ray). Avilés-Monreal et al. [61] obtained ZnO:Na nanophosphors with more than 200 times the sensitivity to β particles than ZnO, achieving linear measurement of β particles at a dose of 512 Gy.

ZnO has two main emission regions: one is NBE, with an emission wavelength of 380 nm, due to the decay of excitons, and a NBE luminous decay time of about a subnanosecond, the other is deep level emission, also known as DBE, with an emission wavelength of 430~800 nm [3]. The main deep defects include oxygen vacancy, zinc vacancy, oxygen gaps, zinc gaps and oxygen inverse sites. The decay time of DBE is much slower than that of NBE. Therefore, in applications that require rapid response, the impact of DBE should be reduced. Norek et al. [62] summarized four ways to enhance the NBE of ZnO, namely synthesis methods and post-synthesis treatment, atomic doping, application of various coating materials and plasma enhancement. Annealing can reduce oxygen vacancy and increase NBE. Interstitial hydrogen doping can be trapped in the oxygen vacancy, thus passivating the receptor levels. Passivation of acceptor levels was confirmed as particularly important for obtaining high NBE efficiency. For nanostructured ZnO, most of the defects are located on the surface, so suitable coatings are also used to block or remove carrier traps to enhance NBE. The low luminescence efficiency of ZnO nanostructures can be significantly improved by the application of metallic nanoparticles or regularly texture metallic substrates which generate plasmonic resonance in a given spectral region. By annealing ZnO:Ga arrays in a 10% hydrogen–argon mixture at 550 °C, Li et al. [63] greatly enhanced the NBE with a decay time of less than 1 ns, and inhibited the DBE with a slow decay time. Li et al. [64] found that covering the surface of ZnO nanostructures with a thin metal layer (such as Zr, Hf, Zn) that can form ohmic contact with the metal and ZnO interface can enhance the ultraviolet NBE of ZnO.

### 3.1. X-ray Detection

**Figure 5:**
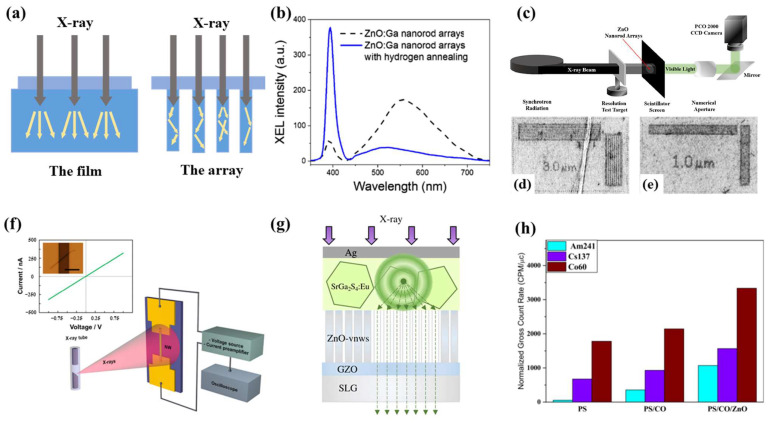
(**a**) Schematic diagram of scintillation photon paths: lateral spreading induced the image blur for the film scintillation screen and scintillation photons guided in designed scintillation screen with nanoarray structural. Typical XEL spectra of the (**b**) ZnO:Ga nanorod arrays unannealed and annealed at 550 °C for 60 min with 20% hydrogen [39]. (**c**) Synchrotron radiation X-ray imaging measuring device schematic, captured microresolution X-ray images and recorded using a PCO2000 camera with (**d**) 10× and (**e**) 20× microscope objectives [39]. (**f**) Schematic diagram of experimental device and the dark state I–V characteristics of ZnO single nanowire devices [30]. (**g**) Schematic diagram of photoconductive effect of ZnO-VNWS in SrGa_2_S_4_:Eu/ZnO-VNWS bilayer [45]. (**h**) Gross count rate per minute per mc (CPM/μc) of the fabricated scintillators (PS), (PS/BECV-DHF) and (PS-BECV-DHF/ZnO) [60].

Due to their high energy, X-rays have a low absorption rate and large penetration depth in most materials and are difficult to detect. In order to realize X-ray detection, many strategies have been explored, including use of G-M counter tubes, scintillators, microchannel plates, charge-coupled devices and silicon drift detectors, etc. [65]. If using scintillator detectors, an excellent scintillator should have a high subsequence number, high light yield, fast decay time and strong radiation hardness. For ZnO, the light yield is not superior to that of perovskite (ZnO: ~2000 photons/MeV, perovskite: >10,000 photons/MeV), but ZnO has subnanosecond NBE and nanosecond DBE, which has great potential in some applications requiring fast decay time. In addition, ZnO also has high radiation hardness and has little influence on the morphology and optical properties under X-ray irradiation [66]. Li et al. inhibited DBE by hydrogen annealing and improved the strength of NBE (Figure 5b). Norek et al. [62] summarized methods for enhancing NBE, including synthesis and post-synthetic processing, atomic doping, surface coatings and surface plasmon resonance. The spatial resolution is an important parameter in the application of X-ray imaging. Transparent scintillation film is a method to obtain high spatial resolution X-ray imaging [67]. However, it still faces the shortcomings of low luminous efficiency and requires a large dose of X-rays. Another way to improve spatial resolution is to build a pixel scintillation layer [68,69]. Figure 5a shows the propagation paths of flicker light in two different structures. It can be found that the array structure can reduce optical crosstalk between adjacent flashing lights and thus improve spatial resolution. Samarin et al. [70] simulated the spatial resolution of the zinc oxide nanowire X-ray imager in anodized aluminum oxide film using MCNP and OPTICS codes on the Geant4 platform, and the results showed that the spatial resolution of the nanowire structure was less than 1 μm. Ming et al. [71] used ion implantation to inject helium ions to a certain depth of ZnO, and used the refractive index waveguide effect to limit the lateral propagation of light, which is also a potential method to improve spatial resolution. Table 5 shows the type of detector used for X-ray detection, as well as structure and peculiarities.

Li et al. [39] prepared hydrothermal ZnO:Ga nanoarrays, which achieved a spatial resolution of 1 μm under X-ray excitation at 20 keV (Figure 5c–e). This is the highest resolution to date using ZnO:Ga for X-ray imaging applications. In other work, the UV emission of ZnO:Ga was enhanced by adjusting annealing temperature, concentration, time and plasma treatment [34,61,72]. The enhancement of NBE is attributed to the fact that hydrogen enters the lattice and occupies oxygen vacancy as the shallow donor, thus inhibiting the DBE and reducing the self-absorption of ZnO. Edgars et al. [30] used the atmospheric pressure chemical vapor transport method to synthesize CdS, SnO_2_ and ZnO nanowires in a horizontal quartz tube reactor and tested the X-ray beam-induced current with the device (Figure 5f). The scale corresponds to a single nanowire device on a Si/SiO_2_ substrate irradiated with X-ray produced by a tungsten anode X-ray tube of 7 μm, and the X-ray beam-induced current is measured by connecting the device to a low-noise current preamplifier and an oscilloscope. Figure 5f shows the I-V curve, which is basically linear, and the optical microscope image of nanowires lying on the electrode. Hikaru et al. [45] used the optical waveguide effect of the array to prepare SrGa_2_S_4_: Eu/ZnO-VNWS bilayer scintillator by electrodeposition (Figure 5g). The spatial resolution of the bilayer scintillator could reach 6.8 μm. Xu et al. [73] formed a direct conversion X-ray detector by sputtering 300 nm MgZnO on a quartz substrate by magnetron sputtering. The function relationship between the net photocurrent (I_p_-I_d_) of the detector under X-ray irradiation and the incident dose rate is basically linear. Device IV in this work exhibited a high sensitivity of 90.9 nC/(Gy_air_·cm^2^) and a short response time of ~0.2 s (rise)/~0.3 s (decay) at 30 V under a 6 MV hard X-ray dose rate of 100 mGy_air_/s. Liang et al. [66] deposited 300 nm thick polycrystalline ZnO films by RF magnetron sputtering technology, and realized an X-ray detector with adjustable sensitivity by precisely controlling the oxygen flux during sputtering. Different oxygen fluxes introduce different amounts of oxygen vacancy, which affects the annihilation rate of photon-generated carriers and the photocurrent intensity of the device. In short, scholars are pursuing X-ray detectors with higher spatial-resolution, more sensitive and more flexible application scenarios.

**Table 5:** Type of detector used for X-ray detection, as well as structure and peculiarities.

Probe Objects and Device Types	Structures and Materials	Peculiarities	Reference
X-rays, scintillator	Nanoarray, ZnO; ZnO:Ga	1 μm spatial resolution; the average attenuation time of ultraviolet emission of ZnO:Ga nanorods array is 37.3 ps, respectively	[39]
X-rays, scintillator	Nanoarray, ZnO:In	The spatial resolution of ZnO:In system is 1.5 µm, its MTF is 377 lp/mm, ultraviolet emission attenuation time is less than 1 ns	[74]
X-rays, scintillator	Nanoarray, ZnO	The optimal spatial resolution of ZnO nanowires with a thickness of 2 μm is 9.8 lp/mm under the irradiation of a 30 keV X-ray beam	[75]
X-rays, scintillator	Nanofilm, ZnO	Adjustable sensitivity is realized by regulating the oxygen flux during sputtering	[65]
X-rays, scintillator	Nanoarray, ZnO	The time constant of exciton luminescence decay time is about 1.1 ns	[48]
X-rays, TSL	Nanofilm, ZnO/Ag/ZnO	The sensitivity is 1.49 times that of TLD-100	[76]
X-rays, scintillator	Nanowires, SrGa_2_S_4_:Eu; ZnO	It emits visible light with a wavelength of 533 nm, a lifetime of 0.389 μs, and a spatial resolution of 6.8 μm	[45]
X-rays, Ionization detector	Nanowires, ZnO	/	[30]
X-rays, Ionization detector	Nanofilm, ZnO:Mg	The response time is about 0.2 s (rise time) and 0.3 s (decay time)	[73]
X-rays, Radiation damage detector	Nanocomposites, CuO; ZnO; PVA; Glycerin plasticizer	When the concentration of nanoparticles is 5%, the response to X-ray is the highest	[54]

### 3.2. γ-ray Detection

γ-rays are the rays released when the nuclear energy level transition is degraded, with strong penetration and damage to cells, which can be used in industry for flaw detection or automatic control of the assembly line, and in medical treatment of tumors. At present, in addition to the direct detection of gamma rays with scintillators, it is more common to measure the ray dose by testing the optical properties and structure changes of ZnO materials before and after gamma radiation. The main parameters of the change are grain size, band gap, emission peak and other parameters. Table 6 shows the type of detector used for γ-ray detection, as well as structure and peculiarities.

Ramakrishnan et al. [77] prepared polyaniline-ZnO thin films by the spin coating method, and tested their optical properties and structure changes after irradiation with a low dose of gamma rays, so as to achieve the purpose of γ-ray dosimetry. Reyhani et al. [33] used SEM, XRD, PL and other characterization methods to study the effects of γ-ray on the optical properties, quality and structure of ZnO nanowires with different diameter. For the irradiated ZnO nanowires, the particle size increases, the stress decreases and the crystallization direction (100) related to Zn metal is generated. The difference of layer transmission spectrum decreases, and the radiation intensity of the green and yellow bands increases compared with the emission intensity near the band edge, which may be caused by the content of Zn metal and the formation of oxygen vacancy. Asemi et al. [60] prepared a PS/BECV-DHF/ZnO sample, which showed a reasonable (CPM/μC) of counts up to 1500 CPM/μC for ^137^Cs and up to 3300 CPM/μC for ^60^Co (Figure 5h).

**Table 6:** Type of detector used for γ-ray detection, as well as structure and peculiarities.

Probe Objects and Device Types	Structures and Materials	Peculiarities	Reference
γ-rays, scintillator	Nanocomposites; PS; BECV-DHF; ZnO	CPM above 3300 for the ^60^Co γ source	[60]
γ-rays, TSL	Nanopowders, ZnO:Y	TL spectra show two broad luminescence peaks: 100 °C (unstable) and 250 °C (stable peak)	[78]
γ-rays, radiation damage detector	Nanoarray, ZnO	ZnO has high radiation hardness under high dose ray (^60^Co) radiation environment	[79]
γ-rays, radiation damage detector	Nanofilm, polyaniline, ZnO	The grain size increases and the band gap decreases with the increase of radiation dose.	[77]

### 3.3. α Particle Detection

α particles are positively charged particles, which are emitted by the decay of certain radioactive materials and made of two neutrons and two protons (He^2+^). Radioactive contamination from a nuclear reactor accident or the explosion of an alpha transmitter’s radiation dispersal device can cause irreversible damage to human internal organs. In addition, sites such as laboratories, nuclear treatment facilities and waste treatment facilities that use alpha sources must be limited below certain permitted levels. There is therefore a need to monitor alpha radiation in emergency management and occupational radiation protection. The time and position mapping of the neutrons produced by the D-T reaction and the α particles produced in the opposite direction of the neutrons is a new technique to determine the direction and time of the produced neutrons, and this method of adjoint particle measurement requires an efficient alpha detector with a fast response [80,81]. Table 7 shows the type of detector used for α-particle detection, as well as structure and peculiarities.

**Figure 6:**
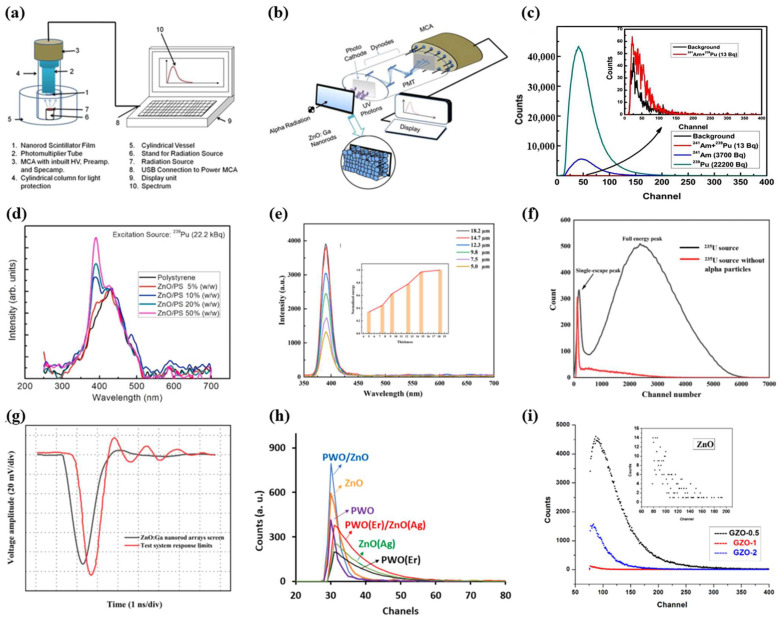
(**a**) Schematic diagram of a prefabricated alpha radiation detector. (**b**) Enlarged view of the detection unit during alpha detection; (**c**) recorded pulse height spectrum for different alpha sources [80]. (**d**) Radioluminescence obtained by ^239^Pu alpha radiation excitation [82]. (**e**) The emission spectra and integral intensity normalized graph of ZnO:Ga nanorod arrays with different thicknesses excited by the ^241^Am alpha particle source. (**f**) Pulse height spectra and (**g**) the time response waveform of hydrogen-annealed ZnO:Ga nanorod arrays excited by ^235^U alpha particle source [40]. (**h**) Pulse height spectrum of the prepared nanocomposite films [83]. (**i**) Pulse height spectra for ^241^Am alpha particles’ response for ZnO:Gd NPs [49].

Sahani et al. [82] prepared ZnO/PS composite film samples by a simple process. The pulse height for ^239^Pu increases with the increase of ZnO particles in PS. When the mass fraction of ZnO/PS is 50%, the detection efficiency is the highest (Figure 6d), about 23%, and MDA < 0.4 Bq. They used the ZnO:Ga nanoarray scintillator prepared by the low-temperature hydrothermal method to achieve an α particle detector (Figure 6a,b) [80]. Figure 6c shows the pulse spectra of ^241^Am + ^239^Pu, ^241^Am and ^239^Pu recorded by the alpha radiation detector from three different sources. The detector has a detection efficiency of about 28.5% and an MDA of 1.7 Bq at a distance of 5 mm from the ^241^Am source detector. The detector has a good reproducibility in the range of ±1%. The ZnO:Ga scintillation screen prepared by Chen et al. [40] has the potential to be applied to APNG. Among them, it can be seen from Figure 6e that the array of 14.7 μm can almost completely deposit the energy of the α particles. The pulse height spectrum of ^235^U shown in Figure 6f shows a good signal-to-noise ratio, which can distinguish between γ signals in the environment. Figure 6g shows the pulse time response waveform of the scintillation screen. The rise time of the ZnO:Ga nanorod array is less than 1 ns, which is close to the natural response limit of the tested system, and the decay time is about 2 ns. Alamdari et al. [55] introduced Ga into a ZnO structure to enhance the interaction between nanoparticles and α particles, which improved the scintillation light yield. They also investigated the effect of composite films of different thicknesses on the counting rate, with thinner films reducing the interaction with α particles and thicker films allowing a large portion of the α energy to be absorbed by the polymer layer. Hosseinpour et al. [83] prepared the PWO (Er)/ZnO (Ag) flexible nanocomposite sensor, which has good stability, repeatability and linear response to alpha radiation (99.6%) (Figure 6h).

**Table 7:** Type of detector used for α-particle detection, as well as structure and peculiarities.

Probe Objects and Device Types	Structures and Materials	Peculiarities	Reference
α particles, scintillator	Nanoarray, ZnO; ZnO:Ga	The α particle response of different growth times was studied	[41]
α particles, scintillator	Nanoarray, ZnO:Ga	The detection efficiency is 28.5%, with good repeatability in the range of 1%	[80]
α particles, scintillator	Nanoarray, ZnO:Ga	The rise time of ZnO:Ga nanorods array is less than 1 ns, and the decay time is about 2 ns	[40]
α particles, scintillator	Nanocomposites, ZnO/ZnO:Ga; PS	The novel and strong blue emission has a counting efficiency of about 50% for α particles from ^241^Am sources	[55]
α particles, scintillator	Nanocomposites, ZnO; PS	MDA < 0.4 Bq	[82]
α particles, scintillator	Nanocomposites, PWO:Er; ZnO:Ag	It has high sensitivity to alpha radiation (99.6%) and exciton lifetime τ = 143.30 ± 0.07 μs	[83]
α particles, scintillator	Nanoparticles, ZnO:Gd	For ^241^Am, the counting efficiency of 0.5%Gd doping is 56.68%, and the response time to FWHM is about 650 ns	[49]

### 3.4. β Particle Detection

β particles are electrons whose mass is very small and their speed can reach the speed of light 9/10. For the detection of β particles, TL and OSL detection methods can be used in addition to scintillation. TL is the light emitted by a previously irradiated solid when heated, and OSL is the light emitted by a previously irradiated solid when illuminated. If the light emission is recorded, it can be used to estimate the radiation dose to which the solid is exposed. Table 8 shows the type of detector used for β-particle detection, as well as structure and peculiarities.

**Figure 7:**
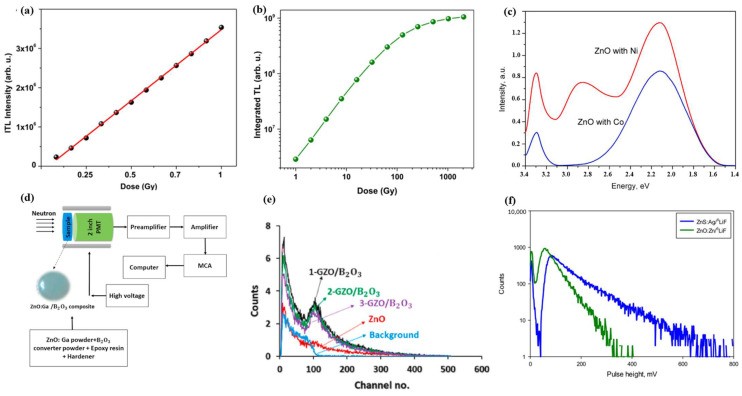
ITL of synthesized ZnO:Na samples as obtained after exposure to different doses of beta particle irradiation in the dose range (**a**) from 0.08 to 1.0 Gy, and (**b**) from 1.0 to 2048 Gy [83]. (**c**) CL spectra of the samples obtained from Zn(NO_3_)_2_ solution containing 0.05 M Ni(NO_3_)_2_ and Co(NO_3_)_2_ [85]. (**d**) The scintillation measurement setup of ZnO:Ga epoxy composite and (**e**) pulse height spectrum [57]. (**f**) Pulse height spectra for ZnS:Ag/^6^LiF and ZnO:Zn/^6^LiF exposed to neutrons and gamma from the moderated ^241^Am–Be source [86].

Avilés-Monreal et al. [61] prepared ZnO:Na nanophosphors by a controlled chemical synthesis method and investigated TL for doses below 1 Gy under irradiation with β particles from 0.08 to 2048 Gy, observing a linear response as a dose function (Figure 7a). For higher doses, linear response is obtained under 512 Gy; for larger doses, followed by saturation behavior (Figure 7b), the TL sensitivity of ZnO:Na is more than 200 times of ZnO.

**Table 8:** Type of detector used for β-particle detection, as well as structure and peculiarities.

Probe Objects and Device Types	Structures and Materials	Peculiarities	Reference
β particles, TSL	Nanophosphors, ZnO:Na	The sensitivity of a TLD-100 dosimeter is 0.02	[61]
β particles, TSL/OSL	Nanophosphors, ZnO	MDD is (492 ± 40) μGy	[84]
β particles; scintillator	Nanoparticles, ZnO:Gd	For ^207^Bi, the counting efficiency of 1%Gd doping is 0.235%	[49]
β particles, scintillator	Nanofilm, ZnO:Ni; ZnO:Co	High concentration of Ni and Co doping up to the degenerate level can improve the PL and CL efficiency of NBE	[85]

### 3.5. Neutron Detection

In fact, because the neutron is not charged, the reaction cross section of the interaction with the material is very small; that is to say, ZnO-based scintillators cannot directly interact with the neutron, so the use of conversion materials is necessary, such as ^10^B, ^10^B_4_C, ^6^Li or ^6^LiF, which are materials with a large neutron absorption cross section. As noted by Ghamsari et al. [87], the key limiting flaw of solid-state neutron scintillators is their inherent γ-ray sensitivity, which makes it difficult for them to detect neutrons in the ubiquitous γ-ray background. In addition, typical neutron gas detectors, such as ^3^He, are very expensive due to their lack of isotopes. Therefore, more economical and simpler neutron detectors are needed, and ZnO can be doped with many different target nuclei, which provides a unique opportunity to produce new neutron detectors [57]. Table 9 shows the type of detector used for neutrons detection, as well as structure and peculiarities.

Alamdari et al. [57] prepared a composite scintillator by suspending a phosphor mixture of B_2_O_3_ and ZnO:Ga in an epoxy matrix, and measured the scintillation response of the ZnO:Ga epoxy composite with an Am-Be neutron source (Figure 7d). In Figure 7e, where 1-ZnO:Ga/B_2_O_3_, 2-ZnO:Ga/B_2_O_3_ and 3-ZnO:Ga/B_2_O_3_ represent B_2_O_3_ powders with mass ratios of 0.2, 0.5 and 1 wt.%, respectively, it can be seen that the composite exhibits a good neutron reaction cross section. When neutrons enter such a detector, recoil protons are generated in the composite, and the resulting photons strike the ZnO:Ga particles and emit light, which passes through the material and eventually reaches the PMT window. Sykora et al. [86] studied ZnO:Zn/^6^LiF as a low-afterglow alternative to ZnS:Ag/^6^LiF. The basic scintillation characteristics of ZnO:Zn are studied and discussed. The pulse-shape distinction between neutrons and γ-rays is explored and utilized by a simple single-photon counting method. A detector that achieves neutron scattering is further realized by fiber coupling ZnO:Zn/^6^LiF to PMT. In preliminary studies of this fiber coupling structure, a ^60^Co sensitivity of ~7 × 10^−6^ has been shown, and the counting rate capability of the ZnS:Ag/^6^LiF-based neutron detector has been demonstrated to be at least six times better (Figure 7f).

**Table 9:** Type of detector used for neutrons detection, as well as structure and peculiarities.

Probe Objects and Device Types	Structures and Materials	Peculiarities	Reference
Neutrons, scintillator	Nanocomposites, ZnO:Ga; ^10^B_2_O_3_; EP	Am-Be light source has good neutron radiation sensitivity and significant blue emission peak (~485 nm)	[57]
Neutrons, scintillator	Nanocomposites, ZnO-^6^LiF; PS	The neutron radiation measurement has a sensitivity of ~203 counts/μSv	[88]
Neutrons, scintillator	Nanocomposites, ZnO:Zn; ^6^LiF	^60^Co gamma sensitivity of ~7 × 10^−6^ is shown and improvements in count rate capability of at least a factor of 6 over ZnS:Ag/^6^LiF-based neutron detectors are demonstrated	[86]

## 4. Summary and Prospects

Thanks to the ultrafast luminescence decay time characteristics of nanostructured ZnO materials and the unique optical properties of nanostructures, they can give ultrafast detection responses to a certain amount of radiation sources. In terms of preparation and structure, hydrothermal methods and electrodeposition methods can be used to synthesize high-quality nanoarrays as high-spatial-resolution X-ray and α particle detection, and sol-gel and co-precipitation methods can be used to synthesize nanocrystals/powders and then composite them to adapt to more complex scenarios. Although ZnO itself has sufficient advantages, there are still some shortcomings that need to be overcome and remedied to improve the detection efficiency of high-energy rays and particles, such as reducing the self-absorption of ZnO itself to improve the luminous efficiency, and controlling defects, impurities, particle size, band gap structure, shallow energy levels and excitation values. In terms of mechanism, the growth mechanism of nanorods/wires and their relationships with luminescence properties need to be further studied. In composite applications, the presence of binders (such as polymers) is thought to affect the performance of scintillators, and improving the scintillation performance of nanocomposites is a necessary condition for expanding their applications. The effect of the thickness of the composite film on the optical output efficiency, cutoff capability and spatial resolution also needs to be considered. Further optimization is needed in detection efficiency, count rate and γ-ray suppression in neutron detection.

## Abbreviations

HPGe	high-purity germanium radiation detector
PET-CT	Positron emission computed tomography scanning technology
XFEL	X-ray free electron laser
VNWS	Vertical nanowires
HMTA	Hexamethylenetetramine
BGO	Bismuth germanium oxide
TEA	Triethanolamine
PVT	Polyethylenetoluene
PS	Polyesters
PDMS	Polydimethylsiloxane
EP	Epoxy epoxide
PVA	Polyvinyl alcohol
TL	Thermoluminescence
OSL	Optically stimulated luminescence
PMT	Photo multiplier tubes
CCD	Charge-coupled devices
NBE	Near-band edge emission
DBE	Defect emission
UV	Ultraviolet
MCNP	Monte Carlo N Particle Transport Code
MTF	Modulation transfer function
TLD	Thermoluminescence detector
XEL	X-ray excitation luminescence
BECV-DHF	1,4-bis (9-ethyl-3-carbo-vinyl)-9,9-dihexylfluorene
CPM	Count rate
APNG	Associated particle neutron generator
MDA	Minimum detectability
PWO	PbWO_4_
FWHM	Full width at half maxima
MDD	Minimum detectable dose
ITL	Integrated thermoluminescence
PL	Photoluminescence
CL	Cathodoluminescence
